# Clinical study on acupuncture acupoints around the eyes in treating myopia in children and adolescents: A study protocol

**DOI:** 10.1097/MD.0000000000022659

**Published:** 2020-10-23

**Authors:** Qun Huang, Yang Yang, Hui Huang, Yanlin Zheng, Wanjie Wang, Tingting Liao, Xili Xiao, Jing Wang, Weiwen Zou, Juan Wang

**Affiliations:** a Department of Ophthalmology; b Department of Respiratory Medicine; c Department of Endocrinology, Hospital of Chengdu University of Traditional Chinese Medicine, Chengdu, China.

**Keywords:** acupuncture, myopia, protocol, randomized controlled trial

## Abstract

**Introduction:**

Myopia is the most common cause of avoidable visual impairment worldwide, which causes huge economic burden and social burden. There are several ways to treat and reduce myopia, but all have drawbacks; this reality drives us to search for additional effective and low-risk interventions of treatment for myopia. Acupuncture is an ancient therapy with a history of thousands of years and is now widely used in the medical system. Some randomized controlled trials have reported that acupuncture, as an adjuvant therapy, can effectively improve the diopter and vision in the sense of myopic children. Deqi is a long-standing belief to ensure the efficacy of acupuncture in the treatment of myopia, but this belief has not been confirmed by sufficient evidence of randomized controlled trials.

**Methods:**

This clinical study is a parallel-group, randomized controlled, and single blind study. Three hundred eligible adolescents will randomly be divided into acupuncture Deqi group, acupuncture without Deqi group, and waiting list group. All groups will be given frame glasses for corrective treatment; patients in the acupuncture Deqi group will be treated with acupuncture at acupoints around the eyes and flat puncture to Deqi, while acupuncture without Deqi group will not flat puncture to Deqi. The waiting list group will not receive acupuncture treatment. The primary outcome will be diopter measurement. Adverse events and safety indexes will be recorded throughout the study.

**Discussion:**

Our study will compare acupuncture Deqi with acupuncture without Deqi, and place it in a control group for the treatment of myopia. The results of this trial are expected to provide solid evidence for the effectiveness and safety of acupuncture combined with Deqi in the treatment of myopia, and hope to provide a reference for clinical practice. The primary outcome will be diopter measurement of the patients before treatment.

**Trial registration:**

ChiCTR2000037874, registered September 3, 2020.

## 1 Introduction

Myopia is the most common cause of avoidable visual impairment worldwide.^[1,2]^ Young academics in South-East Asia now face a frequency of up to 95.5% of myopia,^[3]^ accompanied by a high prevalence of high myopia (10–20%).^[4]^ In 2000, there were 1406 million myopia patients and 163 million high myopia patients, and it is predicted that by 2050, there will be 4758 million myopia patients (49.8% of the world population).^[5]^ Myopia brings further vision challenges, as high myopia increases the risk of pathologic ocular changes such as cataract, glaucoma, retinal detachment, myopic macular degeneration, and myopic choroidal neovascularization, all of which can cause irreversible vision loss.^[6,7]^ The global potential productivity loss associated with the burden of vision impairment was estimated at US$244 billion from uncorrected myopia and US$6 billion from myopic macular degeneration.^[8]^

The multifactorial mechanism of the increased prevalence of myopia is not fully understood. Several theories have been proposed to explain the recent increase and its earlier onset in children, including genetic markers,^[9,10]^ increased near work,^[11]^ and less time spent outdoors.^[12,13]^ There has been an increase in efforts to slow the progression of myopia, and a variety of treatment methods have been recommended for the management of myopia. Conventional therapies included single vision spectacle lenses, multifocal eyeglasses, novel lens eyeglasses, various contact lens therapies, orthokeratology, and muscarinic antagonists such as atropine and pirenzepine.^[14,15]^ However, it is reported that none of the spectacle lenses had any significant effect in slowing the progression of myopia. Gas permeable contact lenses have been shown to slow the progression of myopia, but the low incidence of microbial keratitis should not be dismissed, especially in children. The pharmacological treatment has been reported to be associated with adverse effects, including pupil dilation and temporary paralysis of accommodation.^[16–19]^ Consequently, this reality drives us to search for additional effective and low-risk interventions of treatment for myopia.

Acupuncture is an ancient therapy with a history of thousands of years and is now widely used in the medical system. Some clinical studies and systematic reviews show that acupuncture is increasingly used in the clinical treatment of many eye diseases, including dry eye syndrome,^[20]^ blepharoptosis,^[21]^ oculomotor paralysis,^[22]^ blepharospasm,^[23]^ and other ophthalmic diseases, and has a good effect.^[24,25]^ In addition, some randomized controlled trials (RCTs) have reported that acupuncture, as an adjuvant therapy, can effectively improve the diopter and vision in the sense of myopic children.^[26,27]^ Deqi is considered to be an important parameter to achieve curative effect in acupuncture treatment in traditional Chinese medicine theory and practice.^[28]^ Deqi is a complex cluster of sensation evoked by acupuncture stimulation, including aching, dull, heavy, numb, radiating, spreading, and tingling.^[29]^ Modern neuroimaging monitors the dynamic response of acupuncture with specific regions to the whole brain.^[30–33]^ Compared with sham acupuncture, verum acupuncture at true acupuncture points is more likely to elicit a sense of de qi, and the acupuncture needle sensations of Deqi and sharp pain are associated with different patterns of activations and deactivations in the brain.^[34,35]^ The concept of Deqi is a long-standing belief to ensure the efficacy of acupuncture in the treatment of myopia, but this belief has not been confirmed by sufficient evidence of RCTs. In the current paper, we designed a randomized, single-blind, large-scale clinical trial to compare the curative effect of acupuncture for Deqi and acupuncture without Deqi in adolescent patients with myopia.

## 2 Methods/Design

### 2.1 Ethics and dissemination

The protocols to be used adhere to the principles of the Declaration of Helsinki and have been approved by Chinese Clinical Trial Registry (ChiCTR2000037874). Also, the trial reported in this article is registered by the Chinese Clinical Trial Registry with an identifier of ChiECRCT20200196. Written informed consent will be obtained from each participant before any treatment is given.

### 2.2 Study design

**Figure 1:**
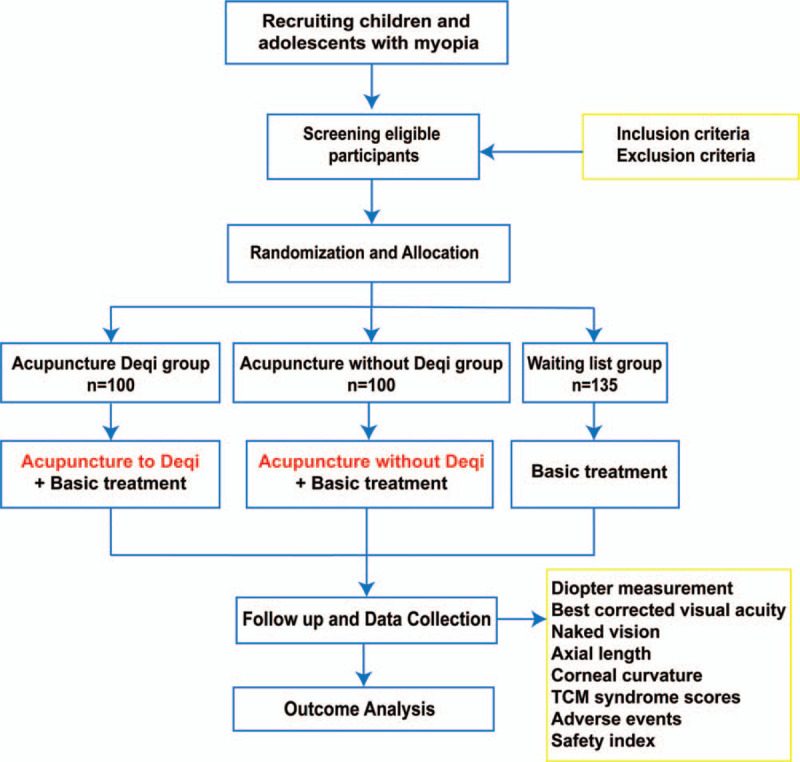
Spirit figure of enrollment, interventions, and assessments. Basic treatment: Conventional frame glasses correction treatment and health education; Safety index: Blood, urine, routine stool, liver function (ALT, AST), renal function (BUN, SCr), and electrocardiogram.

**Figure 2:**
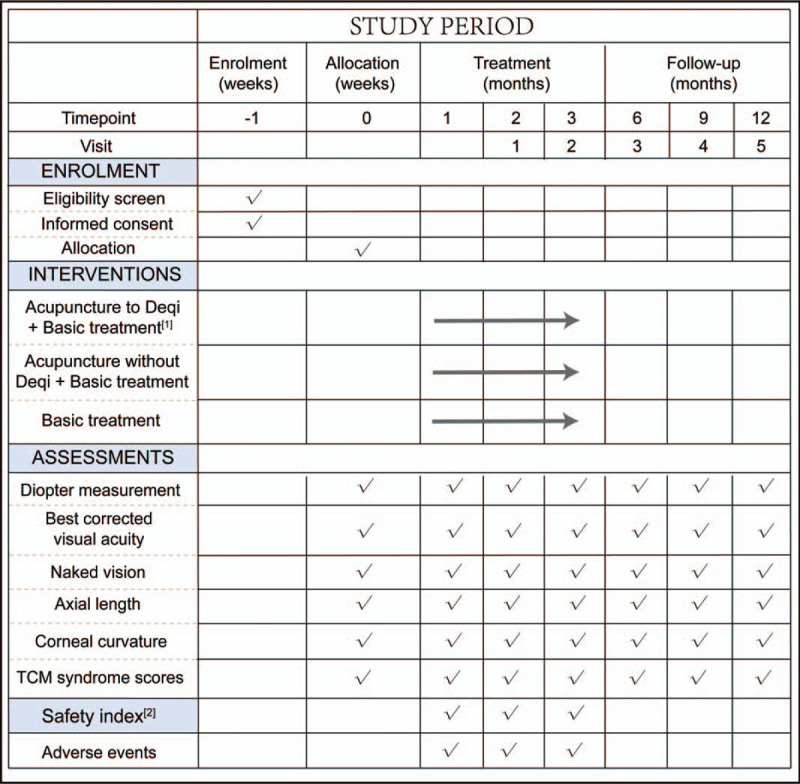
Flowchart of the study design.

This clinical study is a parallel-group, randomized controlled, and single-blind study. The experiment will begin in August 2020 and will be completed in August 2023. After obtaining informed consent, 300 eligible adolescents will be recruited and randomly divided into acupuncture Deqi group, acupuncture without Deqi group, and waiting list group. The trial will include a 3-month treatment period and a 9-month follow-up period. All groups will be given frame glasses for corrective treatment; patients in the acupuncture Deqi group will also be treated with acupuncture at acupoints around the eyes to Deqi, while acupuncture without Deqi group will be treated with acupuncture, but no needle manipulation will be performed. The waiting list group will not receive acupuncture treatment. Results will be assessed at 3 points, including baseline, end of acupuncture treatment, and end of follow-up (Fig. 1 flowchart and Fig. 2 visit indicators). The purpose of this study is to objectively evaluate the clinical efficacy of acupuncture at 3 acupoints (Sizhukong, Tongziliao, and Sibai) in adolescents with myopia. All patients will be required to complete written informed consent before enrolment. If patients are unable to complete written informed consent, it will be obtained from their relatives.

### 2.3 Recruitment strategies

The trial will take place at the Teaching Hospital of Chengdu University of Traditional Chinese Medicine (TCM), China. Through flyers and social media advertisements, participants with myopia will be recruited from the Elementary School near the Chengdu University of Traditional Chinese Medicine. The subjects will be informed of the details regarding the trial plan and those who voluntarily participate in the trial will receive free ophthalmologic examinations. All participants or their guardians have the right to participate or drop out at any time, and will be required to sign the informed written consent before any study procedures.

### 2.4 Diagnostic criteria

IMI's descriptive (qualitative) definition and quantitative definition of myopia are summarized in Table 1.^[36]^

**Table 1:**
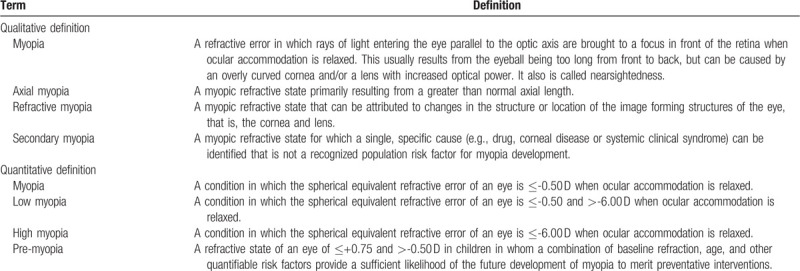
IMI's proposed qualitative definition and quantitative threshold for myopia.

### 2.5 Diagnostic criteria of TCM syndrome classification

Refer to the 2012 editions of *Diagnosis and Efficacy Standards for Diseases and Syndromes of Traditional Chinese Medicine*^[37]^ and *Chinese Medicine Ophthalmology*^[38]^ on the diagnostic basis of “being near and far away,” and combined with clinical practice.

Diagnostic criteria: (liver and kidney deficiency, spleen deficiency syndrome)

Main symptoms: blurred vision, clear vision near;

Secondary symptoms: eye intolerance, dryness and discomfort, distension and pain, photophobia; fatigue, less food, palpitations and forgetfulness, waist and knee acid soft, dizziness tinnitus;

Coating on the tongue: light coating on the tongue;

Pulse: thin or weak; pulse.

This syndrome can be diagnosed by combining the lingual vein with 3 or more secondary symptoms.

### 2.6 Inclusion criteria

Patients with myopia who meet the following inclusion criteria will be enrolled in the trial:

(1) Age of 7∼16 years;
(2) Meet the diagnostic criteria of myopia;
(3) Agree to sign an informed consent form and volunteer for the research.

### 2.7 Exclusion criteria

Patients meeting any of the following criteria will be excluded from the study:

(1) Patients age <7 years or >16 years, patients not suffering from myopia, or patients with myopia and strabismus/amblyopia;
(2) myopia related to retinal dystrophies or collagen syndromes, and developmental disorders;
(3) subjects with serious systemic diseases, such as cerebrovascular, liver, kidney, hematopoietic system, and psychiatric diseases;
(4) use other related drugs or treatments within 2 weeks;
(5) Spherical equivalent (SE) < -0.5D, or combined with pathologic myopia-related fundus changes and/or significant visual function impairment;
(6) the affected eyes have other diseases that affect the determination of acupuncture efficacy;
(7) patients with less than 14 weeks of follow-up, inability to obtain refraction data in the first 14 weeks, patients lost to follow-up, or unable to keep office follow-up at our institution.

### 2.8 Informed consent

Before the study, the general study process and the responsibilities of the participants and researchers will be explained to potential participants or their guardians. Participants or their guardians will be informed that their entry into the trial is entirely voluntary and that they could withdraw at any time. In the event of their withdrawal, data collected on the participant will not be erased and will be used in the final analyses. Written informed consent should be obtained from each participant before he or she undergoes any interventions related to the study.

### 2.9 Sample size

This study is a RCT, and the diopter of the research object is the main observed outcome index. According to previous literature reports, it is estimated that the mean diopter of experimental group 1 is -1.52, the standard deviation is 0.87, and the diopter of experimental group 2 is- 1.81, the standard deviation is 1.25. Set α = 0.25 (2-sided) and β = 0.10. We use PASS 15 software (PASS 15.0.5 NCSS, LLC USA) to calculate the experimental size of the test group and the control group N1 = N2 = N3 = 74 cases. Assuming that the loss to follow-up rate of the study subjects is 20%, the sample size is N1 = N2 =  = N3 = 74÷0.8 = 93 cases. Therefore, the minimum sample size included in this study is 279 cases. In the actual study, a total of 300 cases will be included.

### 2.10 Randomization and allocation concealment

Central random system is used to realize random grouping and random hiding of hierarchical blocks. This study is stratified according to the subcenter, randomly divided into acupuncture Deqi group, acupuncture without Deqi group, and waiting list group at a ratio of 1:1:1. A statistician from Sichuan Evidence-based Medicine Center of Traditional Chinese Medicine will use SAS 9.2 software (SAS, Cary, NC) to generate random sequences in 3 rounds of random sentences, list the random code tables corresponding to the serial numbers 001–300, and generate treatment assignments for 300 subjects. This process is implemented by the online central random system. The researcher enters the screening information of the prospective subjects on the central random system and obtains the subject number. After meeting the subject's criteria and signing the informed consent form, they are randomly divided into groups, and the random number is generated. Examiners and researchers cannot foresee the grouping information of the subjects, and the random number is managed by Sichuan Evidence-based Medicine Center of Traditional Chinese Medicine. Until the end of the study, the subjects, clinicians, and outcome measurers did not know the grouping of subjects.

### 2.11 Blinding

This study uses a single-blind method. Acupuncture and acupuncture pseudo-acupoints will be performed by the same professional acupuncturist in the outpatient department according to uniform standards. All examinations will be checked and evaluated by the same doctor. The subjects will not know the specific grouping situation. Outcome evaluation and data statistics are performed by a third party who is not involved in the treatment. Only in emergency situations, such as a serious adverse event (AE), or when the subject needs emergency rescue, the researcher will report to the supervisor and the principal investigator to decide whether to unblind. Once the subject is unblinded, the case will be regarded as a drop-out case and will not be included in the efficacy analysis. However, if there is an adverse reaction, it should be included in the adverse reaction analysis, and the unblinding should be recorded in the Case Report Forms (CRFs) “Early Subject Exit Page” Related information, including the time of unblindness, reason, treatment, and treatment status.

### 2.12 Intervention

#### 2.12.1 Basic treatment

The basic treatment includes conventional frame glasses correction treatment. All patients will receive disease health education, including eye hygiene, eye use time, rest time, and health consultation, but in order to avoid the confounding effect of excessive eye use, the research team will not provide special eye health intervention measures.

#### 2.12.2 Acupuncture deqi group

On the basis of correction treatment with frame glasses, use acupuncture for acupoint treatment, specific as follows: the patient takes a sitting or supine position, selects eye acupuncture points (Sizukong, Tongziliao, Sibai), local skin and healers who left after routine disinfection 75% ethanol, with the sterile filiform needles (0.25 mm in diameter, 40 mm in length; Hwatuo, Suzhou, China), use both hands to insert the needle, flat puncture to deqi, lift and twist, 20 to 30 min/time.

#### 2.12.3 Acupuncture without deqi group

To avoid the deqi response, no needle manipulation will be performed after the perpendicular insertion. In order to reduce the bias caused by performance, all the rest of the clinical procedures remain the same.

#### 2.12.4 Waiting list group

No intervention will be performed in this group. The participants are required to continue their current living habits, including exercise and diet, while receiving free ophthalmologic examinations throughout the full observational period. To meet the requirements of the Rule of Ethics, all participants in the waiting list group will be offered free acupuncture treatment of their choice at the completion of the study.

#### 2.12.5 Primary outcome

The primary outcome will be diopter measurement of the patients before treatment, 1, 2, 3 months after treatment, and follow-up for 6 , 9 , and 12 months.

Full cycloplegia was obtained 45 minutes after administration of 1% cyclopentolate eye drops (15 mL; Alcon-Couvreur, Puurs, Belgium); Subsequently, the objective optometry (computer optometry and retinoscopy) was measured with a Topcon auto refractor KR8900 (Topcon, Tokyo, Japan); and then subjective optometrics. Subjective optometrics will be divided into the following 2 parts: optometrics will be conducted on a single eye (the right eye followed by the left eye), and a bilateral eye balance examination. Subjective optometrics of a single eye will be divided into the following 3 stages: the initial maximum plus the maximum visual acuity (MPMVA) will be identified, Jackson cross cylindrical lenses will be used to determine the axial direction and degree of the cylindrical lens, and the MPMVA will be repeated. The results will be recorded after optometrics. When the pupils recover, a re-examination will be carried out (3 days after the use of cyclopentolate hydrochloride eye drops). After re-examination, the glasses will be tried again. The degree of the glasses will be marked on the glass trial frame, to be chosen by the subjects based on subjective judgment. Finally, the diopter of the glasses and prescription glasses will be determined. The optometrist will not participate in other parts of the trial.

### 2.13 Secondary outcomes

The secondary outcome indicators are the patient's best corrected visual acuity, naked vision, axial length, corneal curvature, and TCM syndrome scores before treatment, 1, 2, and 3 months after treatment, and at follow-up for 6 months, September, and 12 months.

### 2.14 Safety evaluation

Clinical AEs will be recorded throughout the study. According to previous RCTs, the acupuncture may cause pain, bleeding, hematoma formation, fainting, severe pain, and local infection or other severe discomforts. Any unexpected symptoms or signs during the treatment must be documented regardless of their relation to the study intervention. All details of related and unexpected AEs, such as time of occurrence, severity of AE, and suspected causes, will be recorded on CRFs. Participants with mild and moderate AEs will receive symptomatic treatment and will be closely followed up by the research team. Severe AEs will be reported to the Research Ethics Committee within 48 hours.

Blood, urine, routine stool, liver function (Alanine aminotransferase, ALT, Aspartate aminotransferase, AST), renal function (Blood urea nitrogen, BUN, Serum creatinine concentration, SCr), and electrocardiogram will also be examined before treatment, at 1 month, 2 months and at the end of treatment.

### 2.15 Data management and quality control

All participants are assessed at the first, second, third, sixth, ninth, and twelfth months after recruitment. A well-trained assessor, who is blinded to the treatment assignment, will collect clinical data using CRF files at each visit. Another 2 pre-trained data managers will verify and cross-check the CRFs, for the sake of ensuring reliability and accuracy of the data. If there are any queries in the CRFs, the results will be sent to the third investigator for resolution. Data managers will also be responsible for administration, coordination, and monitoring (including Excel spread sheet-set up, data entry, coding, and query management). Any incomplete data will be coded as unknown, missing, or not applicable, and all data will be anonymously extracted to keep the identities of patients confidential. Results of the analysis must not be released with individual identification of the subject until the Excel spread sheet is closed.

### 2.16 Statistical analyses

Data analyses will be processed with SPSS 22.0 (SPSS Inc., Chicago, IL) and supervised by a skilled statistician blinded to group allocation.

The Full Analysis Set (FAS) is for all participants who have been randomized in terms of the intention-to-treat (ITT), and the per-protocol analysis set (PPS) and is for the individuals who complete the trial and do not have significant protocol violations. On the basis of rule of the last-observation-carried-forward, missing values will be imputed. In order to evaluate therapeutic effect and safety, the FAS and PPS will be used simultaneously.

Categorical data, such as gender and medical history, will be tabulated with frequencies or percentages; and continuous data, such as age and disease course, will be reported as mean ± standard deviation (SD), or median. For the baseline variables, sociodemographic data and other basic indicators will be carried out using analysis of variance (ANOVA) and χ^2^. To compare variables before and after treatment in the same group, a paired *t* test will be used. Repeated-measures ANOVA will be used to compare the intergroup differences among the 3 groups. Tests of ITT between the acupuncture with the deqi group and acupuncture without the deqi group arms and between the acupuncture withou the deqi group and waiting-list group arms with respect to the change will based on time-intervention interactions in the mixed-effect models. A *P* value of less than .05 (2-sided) indicates a statistically significant difference, with 95% confidence intervals.

### 2.17 Monitoring

In the proposed study, a Data Monitoring Committee (DMC) will be installed; the quality control office of the entire project will be set up in DMC. A quality control team will also be set up and a person-in-charge will be appointed [members are the researchers, consisting of personnel from the Ethics Committee, statistical analysis personnel, and clinical professionals (5 people in total)] to identify problems in the project implementation process in a timely manner and to implement the corresponding countermeasures. The collected data will be examined, and the researchers will be supervised to control bias.

## 3 Discussion

Currently, there have been no methodologically controlled clinical studies confirming the efficacy of acupuncture therapy in the treatment of myopia. To meet the demand for high-quality RCTs, our team has designed this randomly controlled trial to explore the potential therapeutic effect of acupuncture on myopia. In this pilot study, we will compare the change in diopter of 3 interventions: acupuncture Deqi, acupuncture without Deqi and waiting list. This 3-arm RCT aims to investigate the relative contributions of the specific (acupuncture Deqi vs acupuncture without Deqi) and the nonspecific (acupuncture without Deqi vs waiting list) effects to the overall clinical effects of the acupuncture Deqi on adolescents with myopia. In particular, a growing body of studies has confirmed that the importance of Deqi, having Deqi, and no Deqi has a huge impact on the experimental structure.

For the purpose of comparing the nonspecific (sham intervention vs no treatment) effects and avoiding bias caused by psychological influences, we set the third group of the waiting-list control without any acupuncture interventions. This is because participants in the acupuncture with the deqi group may have a placebo effect due to the unawareness of group allocation and the treatment that they are undergoing; therefore, placebo effects may happen secondary to their strong beliefs of being in the acupuncture with the deqi group.

To our knowledge, the present study will be the first clinical trial that compares acupuncture Deqi with acupuncture without Deqi, and being placed in a waiting list control group in treating myopia. The scientific and rigorous methodological design of this trial hopefully will provide consolidated evidence on the efficacy and safety of the acupuncture Deqi for treating myopia, and hopefully provide reference to clinical practice Additional file.

## Author contributions

**Conceptualization:** Qun Huang, Yanlin Zheng.

**Investigation:** Hui Huang, Tingting Liao.

**Supervision:** Xili Xiao, Jing Wang, Wanjie Wang.

**Writing – original draft:** Yang Yang, Juan Wang.

**Writing – review & editing:** Qun Huang, Weiwen Zou.

## References

[1] Bourne RR Stevens GA White RA Causes of vision loss worldwide, 1990-2010: a systematic analysis. Lancet Glob Health 2013;1:11. 10.1016/S2214-109X(13)70113-X 25104599

[2] Flaxman SR Bourne RRA Resnikoff S Global causes of blindness and distance vision impairment 1990-2020: a systematic review and meta-analysis. Lancet Glob Health 2017;5:e1221–34. 29032195 10.1016/S2214-109X(17)30393-5

[3] Dolgin E The myopia boom. Nature 2015;519:276–8. 25788077 10.1038/519276a

[4] Morgan IG French AN Ashby RS The epidemics of myopia: aetiology and prevention. Prog Retin Eye Res 2018;62:134–49. 28951126 10.1016/j.preteyeres.2017.09.004

[5] Holden BA Fricke TR Wilson DA Global prevalence of myopia and high myopia and temporal trends from 2000 through 2050. Ophthalmology 2016;123:1036–42. 26875007 10.1016/j.ophtha.2016.01.006

[6] Wong TY Ferreira A Hughes R Epidemiology and disease burden of pathologic myopia and myopic choroidal neovascularization: an evidence-based systematic review. Am J Ophthalmol 2014;157:9–25. 24099276 10.1016/j.ajo.2013.08.010

[7] Ikuno Y Overview of the complications of high myopia. Retina 2017;37:2347–51. 28590964 10.1097/IAE.0000000000001489

[8] Naidoo KS Fricke TR Frick KD Potential lost productivity resulting from the global burden of myopia: systematic review, meta-analysis, and modeling. Ophthalmology 2019;126:338–46. 30342076 10.1016/j.ophtha.2018.10.029

[9] Enthoven CA Tideman JWL Polling JR Interaction between lifestyle and genetic susceptibility in myopia: the Generation R study. Eur J Epidemiol 2019;3:019–0512. 10.1007/s10654-019-00512-7 PMC6602996 30945054

[10] Ghorbani Mojarrad N Williams C Guggenheim JA A genetic risk score and number of myopic parents independently predict myopia. Ophthalmic Physiol Opt 2018;38:492–502. 30182516 10.1111/opo.12579

[11] French AN Morgan IG Mitchell P Risk factors for incident myopia in Australian schoolchildren: the Sydney adolescent vascular and eye study. Ophthalmology 2013;120:2100–8. 23672971 10.1016/j.ophtha.2013.02.035

[12] Guo Y Liu LJ Xu L Outdoor activity and myopia among primary students in rural and urban regions of Beijing. Ophthalmology 2013;120:277–83. 23098368 10.1016/j.ophtha.2012.07.086

[13] Ku PW Steptoe A La YJ The associations between near visual activity and incident myopia in children: a nationwide 4-year follow-up study. Ophthalmology 2019;126:214–20. 29934268 10.1016/j.ophtha.2018.05.010

[14] Huang J Wen D Wang Q Efficacy comparison of 16 interventions for myopia control in children: A network meta-analysis. Ophthalmology 2016;123:697–708. 26826749 10.1016/j.ophtha.2015.11.010

[15] Gong Q Janowski M Luo M Efficacy and adverse effects of atropine in childhood myopia: a meta-analysis. JAMA Ophthalmol 2017;135:624–30. 28494063 10.1001/jamaophthalmol.2017.1091 PMC5710262

[16] Smith MJ Walline JJ Controlling myopia progression in children and adolescents. Adolesc Health Med Ther 2015;6:133–40. 26316834 10.2147/AHMT.S55834 PMC4542412

[17] Weiss RS Park S Recent updates on myopia control: preventing progression 1 diopter at a time. Curr Opin Ophthalmol 2019;30:215–9. 31033732 10.1097/ICU.0000000000000571

[18] Tay SA Farzavandi S Tan D Interventions to reduce myopia progression in children. Strabismus 2017;25:23–32. 28166436 10.1080/09273972.2016.1276940

[19] Cooper J Tkatchenko AV A review of current concepts of the etiology and treatment of myopia. Eye Contact Lens 2018;44:231–47. 29901472 10.1097/ICL.0000000000000499 PMC6023584

[20] Kim BH Kim MH Kang SH Optimizing acupuncture treatment for dry eye syndrome: a systematic review. BMC Complement Altern Med 2018;18:018–2202. 10.1186/s12906-018-2202-0 PMC5934900 29724255

[21] Kim YJ Acupuncture management of blepharoptosis: a case report. Acupunct Med 2020;38:201–2. 31726848 10.1177/0964528419883283

[22] Bi JQ Li W Yang Q Acupuncture for the treatment of oculomotor paralysis: a pilot randomised controlled trial. Evid Based Complement Alternat Med 2016;3961450:24. 10.1155/2016/3961450 PMC4894997 27313646

[23] Bao FF Li QiS Zhang ZY Treatment effects of botulinum toxin injection and acupuncture on blepharospasm assessed by the change in lower eyelid tension. Curr Eye Res 2019;44:679–83. 30724635 10.1080/02713683.2019.1578379

[24] Li TY Xing HJ Xu YY [Features of clinical application of eye acupuncture therapy revealed by data mining]. Zhen Ci Yan Jiu 2019;44:377–82. 31155873 10.13702/j.1000-0607.180495

[25] Zhi F Huang Q Zhao Y [The patterns analysis of clinical application of acupuncture for ophthalmopathy]. Zhongguo Zhen Jiu 2018;38:907–12. 30141305 10.13703/j.0255-2930.2018.08.029

[26] Shang X Chen L Litscher G Acupuncture and lifestyle myopia in primary school children: results from a transcontinental pilot study performed in comparison to moxibustion. Medicines (Basel) 2018;5:95. 10.3390/medicines5030095 PMC6164433 30200316

[27] Li X Zhang H Zhang T [Clinical observation of Zheng's stunt needling technique in the treatment of juvenile myopia]. Zhongguo Zhen Jiu 2018;38:147–52. 29473357 10.13703/j.0255-2930.2018.02.010

[28] Pan Q Ma L Yang Y [Application of data mining on the relationship between deqi and effect]. Zhongguo Zhen Jiu 2017;37:668–72. 29231513 10.13703/j.0255-2930.2017.06.026

[29] MacPherson H Asghar A Acupuncture needle sensations associated with De Qi: a classification based on experts’ ratings. J Altern Complement Med 2006;12:633–7. 16970533 10.1089/acm.2006.12.633

[30] Theysohn N Choi KE Gizewski ER Acupuncture-related modulation of pain-associated brain networks during electrical pain stimulation: a functional magnetic resonance imaging study. J Altern Complement Med 2014;20:893–900. 25389905 10.1089/acm.2014.0105 PMC4270153

[31] Chen AC Liu FJ Wang L Mode and site of acupuncture modulation in the human brain: 3D (124-ch) EEG power spectrum mapping and source imaging. Neuroimage 2006;29:1080–91. 16325429 10.1016/j.neuroimage.2005.08.066

[32] Jin L Sun J Xu Z Intersubject synchronisation analysis of brain activity associated with the instant effects of acupuncture: an fMRI study. Acupunct Med 2018;36:14–20. 29259022 10.1136/acupmed-2016-011327

[33] Takamoto K Urakawa S Sakai K Effects of acupuncture needling with specific sensation on cerebral hemodynamics and autonomic nervous activity in humans. Int Rev Neurobiol 2013;111:25–48. 24215916 10.1016/B978-0-12-411545-3.00002-X

[34] Chen JR Li GL Zhang GF Brain areas involved in acupuncture needling sensation of de qi: a single-photon emission computed tomography (SPECT) study. Acupunct Med 2012;30:316–23. 23023060 10.1136/acupmed-2012-010169

[35] Sun J Zhu Y Jin L Partly separated activations in the spatial distribution between de-qi and sharp pain during acupuncture stimulation: an fMRI-based study. Evid Based Complement Alternat Med 2012;934085:24. 10.1155/2012/934085 PMC3544542 23326294

[36] Flitcroft DI He M Jonas JB IMI - defining and classifying myopia: a proposed set of standards for clinical and epidemiologic studies. Invest Ophthalmol Vis Sci 2019;60:M20–30. 30817826 10.1167/iovs.18-25957 PMC6735818

[37] State Administration of Traditional Chinese, Medicine. Standards for Diagnosis and Efficacy of TCM Diseases and Syndromes. 2012;Beijing: China Medical Science and Technology Press, 93–94.

[38] Zeng QH Chinese Medicine Ophthalmology. 2003;Beijing: China Chinese Medicine Press, 244:148-149.

